# The Biological Basis of a Universal Constraint on Color Naming: Cone Contrasts and the Two-Way Categorization of Colors

**DOI:** 10.1371/journal.pone.0024994

**Published:** 2011-09-21

**Authors:** Youping Xiao, Christopher Kavanau, Lauren Bertin, Ehud Kaplan

**Affiliations:** 1 The Neuroscience Department and the Friedman Brain Institute, Mount Sinai School of Medicine, New York, New York, United States of America; 2 Natural Sciences and Mathematics, Bennington College, Bennington, Vermont, United States of America; Kyushu University, Japan

## Abstract

Many studies have provided evidence for the existence of universal constraints on color categorization or naming in various languages, but the biological basis of these constraints is unknown. A recent study of the pattern of color categorization across numerous languages has suggested that these patterns tend to avoid straddling a region in color space at or near the border between the English composite categories of “warm” and “cool”. This *fault line* in color space represents a fundamental constraint on color naming. Here we report that the two-way categorization along the fault line is correlated with the sign of the L- *versus* M-cone contrast of a stimulus color. Moreover, we found that the sign of the L-M cone contrast also accounted for the two-way clustering of the spatially distributed neural responses in small regions of the macaque primary visual cortex, visualized with optical imaging. These small regions correspond to the *hue maps,* where our previous study found a spatially organized representation of stimulus hue. Altogether, these results establish a direct link between a universal constraint on color naming and the cone-specific information that is represented in the primate early visual system.

## Introduction

Our perceptual color space is continuous. However, each language partitions this space into a modest number of categories, and assigns a basic color term for each category. Since the classic work of Berlin and Kay (1969) [1], it has been intensely debated whether there are universal constraints on how various languages partition color space [2], [3], [4], [5], [6]. This debate has become one of the central topics of a more general controversy in cognitive science, concerning the relation between language and perception [7]. Statistical analyses of the database of the *World Color Survey* (WCS) have provided new evidence for the existence of universal constraints on color naming [2], [3], [8], and recent theoretical work has suggested perceptual, environmental, and social bases for these constraints [9], [10], [11], [12], but their biological basis remained unknown.

In the WCS study [13], an informant was asked to name the color of each one of the 320 selected Munsell color chips. These chips formed a 2-D array representing forty equally spaced hues at eight levels of lightness or Munsell Value, each at maximum available saturation or Munsell Chroma. From these data, the subset of color chips named by each of the basic color terms in the given informant's language was identified. These subsets are called *color-naming palettes* below. The best example of each color term was also identified. Since most informants were from pre-industrial societies with unwritten languages, it is believed that the WCS languages were largely uncontaminated by contact with industrialized cultures that possess English-like color lexicons. If there were universal constrains on color naming across various languages, the color-naming palettes should not be randomly distributed in color space. Statistical analysis of the palette centroids and best examples upheld this prediction [2], [3].

However, the above analysis was focused on the palette centroids and best examples, without examining the full pattern of each palette. To address this issue, Lindsey and Brown [8] represented each color term of an informant with a vector that had 320 elements, one for every chromatic chip. These vectors are called color-naming vectors below. Each element in a color-naming vector has a value of 1 or 0, depending on whether the corresponding chip was included in the corresponding color-naming palette of the particular informant. The collection of these color-naming vectors was subjected to a *k*-means cluster analysis, in which all vectors were assigned to k clusters based on the Pearson correlation-based similarity between vectors.

When *k* = 2 was used, one cluster corresponded to a group of colors that largely fall into the English composite color category “warm”, while the other cluster corresponded to the category “cool”. This border also manifested itself when *k* = 3 to 10 was used in the cluster analysis. All the resulting clusters were encompassed by either the “warm” or the “cool” clusters described above, and none of their represented color regions straddled the “warm/cool” border. Considering that the cluster operations at different k values were independent of each other, the persistence of the “warm/cool” border in their results suggests that this border region is special in color space.

In addition, the same study found that the “warm/cool” border largely coincided with the region in color space where the concordance for color naming was the lowest. The concordance was measured among individuals speaking the same language, and then averaged across all languages tested. Since disagreements on color naming tend to be more frequently for colors that are near the edge of a color-naming palette than for those well inside a palette, the concordance value of a test chip reflects the chance of that chip being located well inside a palette across the database. Taken together, the results of this study suggest that, on average across the WSC languages, the “warm/cool” border region in color space is less likely to pass through the interior of a color-naming palette as compared with other regions. In other words, the “warm/cool” border tends to be a “barrier” or a “fault line” for color-naming palettes [8]. Therefore, this border or the associated two-way categorization of colors imposes a universal constraint on how the palettes of a particular language are distributed in color space.

To determine the biological basis of this universal constraint, we first related the two-way categorization of color chips to the cone excitation they elicit. Specifically, we examined the influence of the two cone-opponent channels in the retinogeniculate pathway, one encoding L- *versus* M-cone contrast (L−M), and the other encoding S- *versus* (L+M)- cone contrast (S-(L+M)) [14], [15], [16], [17]. We found that the two-way categorization predominantly followed the sign of the L−M contrast, with only negligible influence from the S-(L+M) contrast. We then examined cortical responses to color in the macaque primary visual cortex (V1), using imaging of intrinsic optical signals [18]. Our analysis suggest that the two-way categorization of the cortical responses to color stimuli also follows the sign of the stimulus L−M contrast.

## Results

### Two-way categorization of colors and cone excitation

In the study by Lindsey and Brown (2006), *k*-means cluster analysis (*k* = 2) assigned all color-naming palettes in the WCS to either “warm” or “cool” clusters. Since each tested Munsell color chip is present in many color-naming palettes, the above analysis generated a pair of numbers for each color chip, representing the number of times that the given chip was present in a “warm” or “cool” palette. In the following analysis, we denote this pair of numbers as N_w_ and N_c_, respectively. The entire set of N_w_ and N_c_ were generously provided to us by Lindsey and Brown.

For each color chip we calculated an index: I_wc_ = (N_w_−N_c_)/(N_w_+N_c_). The sign of I_wc_ represents the classification of a chip into the “warm” *versus* “cool” category, as deduced from the entire set of color-naming palettes in the WCS. The value of I_wc_ represents the level of consistency of this classification across various palettes.

To relate I_wc_ to activity in the retinogeniculate pathway, we calculated the expected responses to each color chip in the two cone-opponent channels that carry information about color. The response of the L−M channel was given by R_LM_ = 0.5C_L_−0.5C_M_, and the response of the S-(L+M) channel was given by R_Y_ = 0.25C_L_+0.25C_M_−0.5C_S_, where C_L_, C_M_, and C_s_ denote the contrast of the respective cone excitation between a test chip and a gray chip that was also included in the WCS test set. For instance, C_L_ = (L−L_0_)/L_0_, where L and L_0_ stand for the L-cone excitations of the test and gray chips, respectively [19]. The weights of various cone contrasts in R_LM_ and R_Y_ were suggested by a previous study on the lateral geniculate nucleus (LGN) [17]. According to that study, most cells that received opposing L- and M-cone inputs had a weight of close to 0.5 for each of these two cone contrasts. Most cells that received opposing S- and (L+M)-cone inputs had a weight of close to 0.5 for the S-cone contrast. The weights for L- and M-cone contrasts varied substantially across the second group of cells, but were centered near (−0.25, −0.25) or (0.25, 0.25) (Fig. 6 in ref [17]). Our choice of the cone weights for the two cone-opponent channels is also consistent with studies that related cone contrasts with the sensitivity of human color perception or cortical activity [19], [20].

In the following analysis, we used as a reference the gray chip with a lightness value of 5. If we had used a different gray chip as the reference, our results would have remained the same, since different gray chips have nearly identical spectral reflectance, which will scale various cone contrasts by the same factor without altering their relative amplitudes.

**Figure 1:**
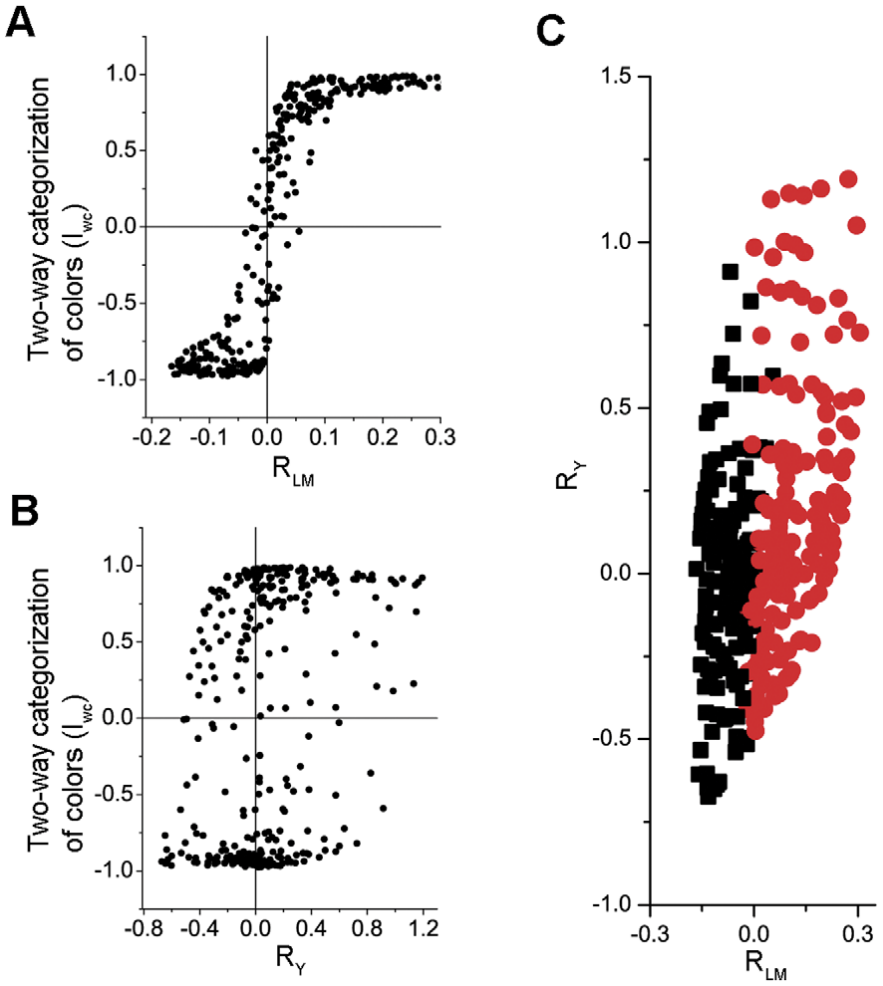
Two-way categorization and cone contrasts of the color chips used in the World Color Survey (WCS). **A–B**, I_wc_ as a function of R_LM_ and R_Y_ respectively. The sign of I_wc_ indicates the category that a chip was assigned to. The value of I_wc_ reflects the consistency of the categorization across the WCS database. R_LM_ and R_Y_ denote the L−M and (L+M)-S cone contrast, respectively. **C**, Scatter plot of R_LM_ *versus* R_Y_. The chips in different categories are represented by symbols of different colors (red *vs*. black).

Figures 1A and 1B show the value of I_wc_ as a function of R_LM_ and R_Y_, respectively. In Fig. 1A, the vast majority of data points (304/320, 95%) fell into the first and third quadrants, where the signs of I_wc_ and R_LM_ were the same. This suggests that the two-way categorization of a color chip can be predicted largely by the sign of the R_LM_ that the given chip elicited. In contrast, in Fig. 1B only 61% (196/320) data points fell into the first and third quadrants, indicating that the sign of R_Y_ was a much poorer predictor of the two-way categorization.

#### SVM analysis

To determine whether the two-way categorization of colors can be better predicted by a linear combination of R_LM_ and R_Y_, we used a linear *Support Vector Machine* (SVM) classifier based on the sign of I_wc_ *versus* the values of R_LM_ and R_Y_ (Fig. 1C). Unlike other classification algorithms, SVM is not based on any assumptions about the sample's probability distribution function, and therefore achieves better classification when the probability distribution is unknown [21]. A linear SVM classifier is represented by a hyperplane that separates two classes of samples in a multi-dimensional space of the input variables.

The SVM-derived hyperplane for our dataset is represented by:
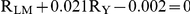



With a 320-fold cross-validation, the accuracy of the linear SVM classification was 95.9%. This high accuracy suggests that the two-way categorization of colors can be approximated by a linear process involving R_LM_ and R_Y_.

One way to determine the relative importance of different input variables in a linear SVM classifier is to compare their w^2^, where w denotes the weight of a variable in the equation that represents the classifier's hyperpane [22]. However, for this purpose, each variable needs to be normalized before an SVM classifier is trained. For the above classifier, the w^2^ for the normalized R_LM_ and R_Y_ were 1 and 0.0045, respectively. The large ratio between these two numbers (221) indicates that this classifier was predominantly based on R_LM,_ with only a negligible influence of R_Y_. In addition, since the hyperplane intercepts with the R_LM_ axis at R_LM_ = 0.002, which is below or close to our perceptual threshold along the L−M direction [19], [20], the SVM classifier suggests that the two-way categorization of colors is predominantly correlated with the sign of R_LM_.

To determine further the contribution of R_Y_ to the two-way classification of colors, we compared the performance of the above classifier with that of another one, which used R_LM_ as the only input variable. To this end, we calculated an information criterion, SVMICb, that was developed by Claeskens et al. (2008) [23] to select the input variables that contribute to SVM-based classification. This creation of criterion was stimulated by the Bayesian information criterion (BIC) that is widely used for model selection [24]. Claeskens et al. (2008) have shown that excluding an input variable that is uncorrelated with the classification tends to reduce the value of SVMICb. But this value would increase if the excluded variable were correlated with the classification, and this correlation was not caused by the correlation between the given variable and another one. Such an input variable is deemed to contribute to the classification. A variable is deemed not to contribute to the classification if it is uncorrelated with the classification, or if such a correlation is caused by the correlation between variables. By comparing the SVMICb values for our two SVM classifiers, one using all input variables and the other excluding only one variable, we can infer whether the excluded variable contributes to the SVM classification.

The classifier with both R_LM_ and R_Y_ as input variables had an SVMICb value of 62.49, while the one with only R_LM_ as the input variable had an SVMICb value of 60.54. This reduction in the SVMICb value with the exclusion of R_Y_ suggests that R_Y_ did not contribute to the linear SVM classification. In contrast, the classifier with only R_Y_ as the input variable had an SVMICb value of 282.29. This substantial increase in the SVMICb value suggests that R_LM_ was a major contributor in the classifier that operated on both R_LM_ and R_Y_, which is consistent with the relative importance of R_LM_ and R_Y_ as measured by their w^2^ in that classifier.

A similar result was obtained when the test colors were represented in the 3-dimensional cone-contrast space, where the axes represent C_L_, C_M_, and C_S_, respectively. Operating in that space, the SVM classification algorithm generated a separating hyperplane given by:
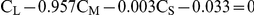



The accuracy of this classifier was 90.31% (based on a 320-fold cross-validation). The weights in this equation also suggest that the classification is predominantly based on the value of C_L_-0.957C_M_, which approximates R_LM_. The SVMICb value of this classifier was 124.79. This value was reduced to 118.64 when C_S_ was excluded from the classification, and the accuracy was increased to 90.63%, suggesting that the S-cone contrast did not contribute to the two-way classification of colors. In contrast, the SVMICb value was increased to 363.20 or 261.41 when either C_L_ or C_M_ was excluded from the classification, consistent with the notion that both L- and M-cone contrasts were major contributors in the original classifier that operated in the 3-dimensional space of cone contrasts.

### Two-way clustering of cortical responses to color and cone excitation

**Figure 2:**
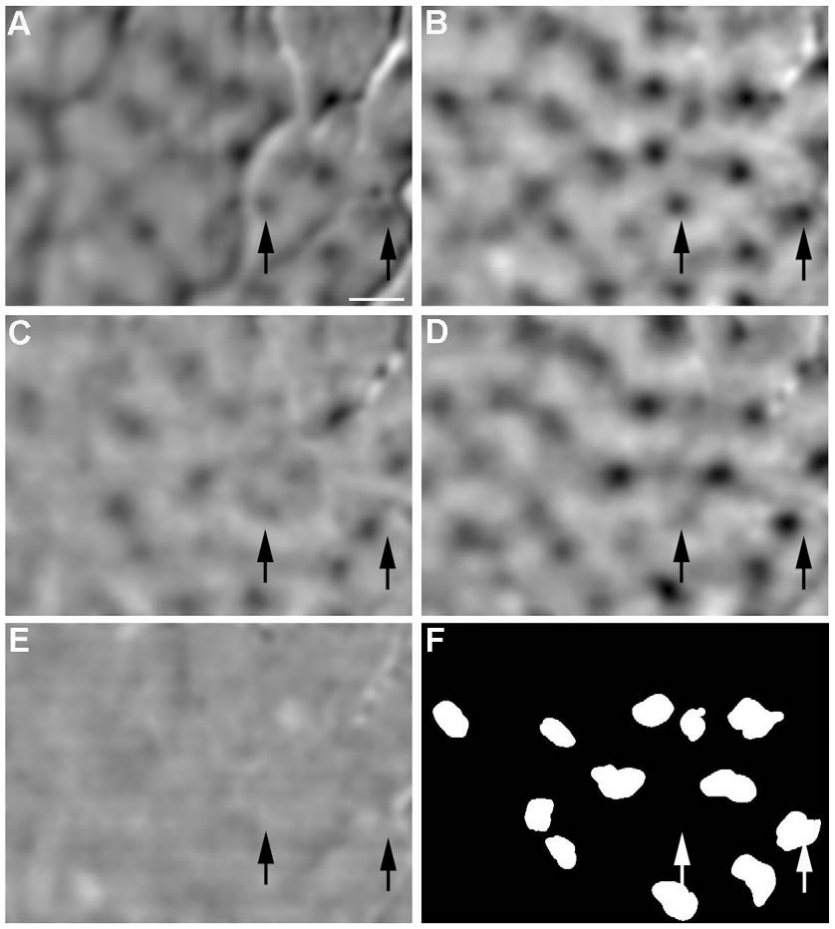
V1 responses to spatially uniform colors. **A**, A conventional activation map in response to a red stimulus. **B–D**, The SVM-derived activation maps in response to red, green, and blue stimuli, respectively. **E**, The SVM-derived activation map from trials without color stimulation (gray screen). **F**, The patches responding significantly to the majority of the tested colors. Scale bar, 0.5 mm.

To determine whether the R_LM_-correlated two-way categorization is reflected in cortical response to color, we reanalyzed the color responses in macaque V1 that we imaged with intrinsic optical signal and reported previously [18]. Unlike the previous study [18], here we used an SVM-based method to derive cortical responses to individual colors, or *single-condition* activation maps [25]. Fig. 2A shows the single-condition map associated with a red stimulus that was calculated with the conventional method of subtraction and normalization [18]. The dark patches represent regions that were activated by the red stimulus. Two of them are indicated by the arrows.

Fig. 2B shows the corresponding map produced by the SVM-based method. The usual artifacts associated with blood vessels in Fig. 2A are largely absent in Fig. 2B, validating the superiority of the new method [22] over the conventional one. Responses to the nine colors that were presented in all experiments were used in this study, and the activation maps associated with green and blue are shown in Fig. 2C–D. The two arrows mark the same pair of locations in all panels. The response patch indicated by the right arrow is readily visible in all maps with slight difference in location for different colors [18]. However, the patch indicated by the left arrow is barely visible in Fig. 2C–D, indicating weak responses in this region to green and blue stimuli. Consequently, the spatial distribution of the responses to green and blue in this region cannot be reliably extracted from the noise that is inherent in optical imaging. It is therefore desirable to exclude from further analysis weakly responsive regions like this one, along with the nonresponsive regions.

To this end, we estimated the magnitude of noise in the activation maps from a control map that is associated with the control trials in which the display was maintained at the adaptation gray. Based on the estimated noise magnitude, a threshold of response was determined and was used to identify cortical *response patches* where responses to more than half of the color stimuli were above the noise magnitude (Fig. 2F, see Methods for details on identifying response patches). In total we analyzed 12 response patches from the hemisphere shown in Fig. 2, and altogether 33 patches from 4 hemispheres. Some of these patches corresponded to the regions where our previous study found a spatially organized representation of hue, or *hue maps* [18] (see Discussion).

**Figure 3:**
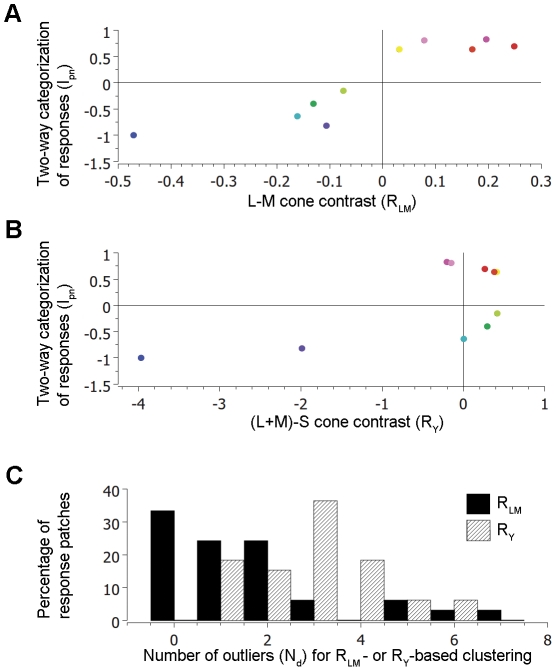
Two-way clustering of V1 responses to color. **A–B**, I_pn_ as a function of R_LM_ and R_Y_, respectively, averaged across all response patches. The sign of I_pn_ indicates the cluster that a response was assigned to, and the value indicates the consistency of the assignment across the response patches. **C**, The distribution of response patches with different number of outliers (N_d_) according to the R_LM_-based clustering (black) or R_Y_-based clustering (striped).

For each response patch, the response to a color was represented by a vector where each element is the value of a pixel within that patch in the corresponding activation map. To study the *spatial distribution* of the responses within a response patch without considering their *amplitude*, each response vector was normalized to have a zero mean and a unit standard deviation. At each patch, the normalized response vectors associated with different colors were divided into two clusters by the *k-*means cluster analysis (*k* = 2). To determine whether this clustering is related to R_LM_ and/or R_Y_, we calculated these values for all tested colors. To compare the results across different response patches, we defined the cluster of response vectors that included the blue-associated one as the negative cluster, since blue had the most negative values of R_LM_ and R_Y_ among all tested colors (Fig. 3). The other cluster of response vectors was defined as the positive one. We then calculated an index for each tested color: I_pn_ = (N_p_−N_n_)/(N_p_+N_n_), where N_p_ denotes the number of response patches where the response to the given color was assigned to the positive cluster, and N_n_ denotes the corresponding number for the negative cluster. A positive I_pn_ indicates that the response to the given color was assigned to the positive cluster more often than it was to the negative one, and the corresponding color was classified as a positive color. A color with a negative I_pn_ was classified as a negative color. The value of I_pn_ reflects the consistency of clustering across all patches.

Figures 3A and 3B show I_pn_ as a function of R_LM_ and R_Y_, respectively. Since the blue-associated response was, by definition, always in the negative cluster, it was excluded from the following statistical analysis.

In Fig. 3A, all data points fall into the first and third quadrants, where the signs of the abscissa and ordinate values were the same. This distribution of points between quadrants 1&3 *versus* 2&4 (9 vs. 0) was unlikely the result of a random process where each quadrant has equal chance (P<0.005, binomial test). This result suggests that the two-way clustering of the cortical responses was correlated with the sign of the stimulus R_LM_.

In contrast, the distribution of points in quadrants 1&3 *versus* 2&4 in Fig. 3B (5 vs. 4) is not significantly different from a random distribution (P = 1, binomial test), suggesting that the response clustering was uncorrelated with the sign of the stimulus R_Y_.

It is noteworthy that when the set of R_LM_ in each experiment was subjected to *k-*means analysis (*k* = 2), the positive and negative R_LM_ were grouped into separate clusters. This result raised the possibility that the two-way clustering of the cortical responses was simply a reflection of the two-way clustering of R_LM_. To test this possibility, we performed the *k*-means analysis on R_LM_ and cortical responses after excluding one test color. For three experiments that used the same set of stimuli, excluding any color did not affect the clustering of R_LM_, and therefore the results cannot be used to test the above hypothesis. But for the remaining experiment that used a different color pink, excluding the color blue resulted in a two-way clustering of R_LM_ that did not follow the sign of R_LM_. The color yellow that has a positive R_LM_ was assigned to the cluster of colors with negative R_LM_ by the *k*-means analysis. However, the cortical response to color yellow was still assigned to the group of responses to positive R_LM_. In other words, the two-way clustering of the cortical responses followed the sign of R_LM_, regardless of how the set of R_LM_ itself was clustered. These results suggest that the clustering of the cortical responses was not simply an artifact caused by the clustering of the stimulus R_LM_.

#### Responses in individual cortical patches

The above results regarding the averaged behavior of all response patches support the notion that the two-way clustering of color responses in V1 follows the sign of the stimulus R_LM_, and is unrelated to the stimulus R_Y_. This conclusion is further supported by an additional analysis that relates the response clustering to stimulus R_LM_ and R_Y_ at individual response patches. At each patch, we counted how many response vectors were inconsistent with the hypothesis that the response clustering follows the sign of R_LM_ or R_Y_. We call this the number of outliers (denoted by N_d_) below, and it can theoretically have a value from 0 to 8.

Fig. 3C shows the distributions of N_d_ for R_LM_ -based clustering (Black) and R_Y_ -based clustering (striped). While the vast majority of patches (27/33, 82%) contained 2 or fewer outliers of the R_LM_-based clustering, only a minority of patches (11/33, 33%) did so with respect to the R_Y_-based clustering. On average, each response patch contained 1.61+/−0.32 (Mean+/−s.e.m.) outliers of the R_LM_-based clustering, which was significantly less than the outliers of the R_Y_-based clustering (2.97+/−0.24, P<0.001, one-tail, Student's *t*-test, n = 33).

## Discussion

### Two-way categorization of colors and perceptual color temperature

A quantitative analysis of the WCS data by Lindsey and Brown (2006) suggested that the two-way categorization of color represents a universal constraint on color naming across various languages. Our analysis of their results suggests that this categorization is predominantly correlated with the sign of the L−M cone contrast of a stimulus. Moreover, we found that responses of V1 to various colors were clustered according to the sign of the L−M contrast as well. Taken together, our results suggest that the sign of the L−M contrast imposes a fundamental constraint on color naming by grouping the responses of V1 to color.

Although Lindsey and Brown (2006) labeled the two categories as “warm” and “cool” based on the authors' personal experience, the relationship between this dichotomy and the perceptual color temperature is uncertain for the following reasons. First, the color temperature of the WCS chips has not been determined experimentally; Second, the perceptual temperature of a color may vary substantially between individual, as suggested by the disagreement among artists over whether orange-red or yellow is the warmest color; Third, in the original publication that introduced the concept of *perceptual* color temperature, purple was assigned to the “cool” category [26]. However, in the two-way categorization derived from Lindsey and Brown's analysis, purple was grouped with typical “warm” colors. Therefore, although our results established a link between the L−M cone contrast and the WCS-derived dichotomy, further investigation is needed to determine the relationship between cone contrasts and the perceptual color temperature.

### Information criteria for variable selection in the SVM classification

In our analysis of the WCS with SVM classification, we calculated a information criterion, the SVMICb, that was developed by Claeskens *et al.* (2008) [23] to select input variables that contribute to a classification. Based on this criterion, we concluded that neither R_Y_ nor C_S_ contributed to the two-way categorization of colors. In addition to this criterion, we calculated another one, the SVMICa, that was also developed by Claeskens *et al.* (2008). The SVMICa value was 113.49 for the classifier with all cone contrasts as input variables, and 111.10 for the one that excluded C_S_. This difference in SVMICa is consistent with that in SVMICb, and corroborates the notion that C_S_ did not contribute to the two-way categorization.

However, these two information criteria seemed to deliver opposite inferences regarding the role of R_Y_. Compared to the classifier with both R_LM_ and R_Y_ as input variables, the one with the exclusion of R_Y_ had a smaller value of SVMICb (62.49 versus 60.54), but a large value of SVMICa (54.95 versus 56.77). This discrepancy was caused by the difference in the term of these criteria that penalizes the number of input variables. This term in the SVMICa and SVMICb is similar to that in the Akaike information criterion (AIC) and Bayesian information criterion (BIC), respectively. The simulation studies in Claeskens *et al.* (2008) [23] suggested that the SVMICb performed much better than the SVMICa in selecting the correct set of variables that contributed to the classification. Although the former presented a small chance of underfitting, namely discarding a contributing variable, the latter presented a much larger chance of overfitting, namely selecting a non-contributing variable as a contributing one. Therefore, we put more weight on the SVMICb-based selection.

### The perceptual and physiological basis of universal constraints on color naming

The major controversy over color categorization is whether there are universal constraints on the location and pattern of each category in color space, in addition to the widely accepted constraint of connectedness, namely, each category must cover a contiguous region of color space [7].

Statistical analyses of the WCS data demonstrated that color categories of most languages are centered around a modest set of locations in color space [2], [3], and thus provided strong evidence for the existence of universal constraints. Computer simulations suggested that this non-random distribution of color categories can be accounted for by the wavelength discrimination function of humans [27]. Since the wavelength discrimination function seems to be partially attributed to the characteristics of the L−M and S-(L+M) channels in the lateral geniculate nucleus [28], that simulation study implies a link between the universal constraints and the functional characteristics of the retinogeniculate pathway.

A model-based study of the WCS data has related the universal constraints to the perceptual distance between colors that is embodied in the CIELAB color space [9]. It suggested that the perceptual distance-based clustering of colors may partially shape all color-naming systems. However, as the authors pointed out, there are many color-naming systems that significantly deviate from the model's prediction, which leaves open the possibility that factors other than the universal constraints are also involved in the formation of color categories of a particular language. Because of these additional factors, such as linguistic convention [4], [6], a universal constraint may only manifest itself in measures that characterize the average properties across many languages. Therefore, while the “warm/cool” border tends to be a barrier in color space for color-naming palettes of most of the WCS languages, as suggested by the results of Lindsey and Brown (2006), some languages may have palettes that cross or straddle this border to a substantial extent.

Although our results point to a prominent role of the L−M contrast in shaping the lexical coding of color, they do not exclude the possibility that the S-(L+M) contrast could play a role in this process under some circumstance, such as in generating the green and blue categories by splitting the “grue” category that is preserved in some languages [29], [30]. Moreover, many universal constraints other than the “fault line” are likely to be caused by mechanisms beyond the early visual system, as suggested by the aforementioned model-based study [9].

We note that 5% of the data points in Fig. 1A fell into the second and forth quadrants, which is inconsistent with the prediction of our hypothesis. However, their corresponding color chips all had low L−M cone contrast (<0.055). These low contrasts could be assigned with wrong signs by our calculation because the calculation was based on the assumption that each WCS experiment was carried out under the CIE standard illuminant D65. While D65 is a good estimate of the average daylight the actual daylight during any WCS experiment was slightly different depending on the time of the day, season of the year, geographic location, and atmospheric conditions [31]. This slight difference in illuminant wavelength is less likely to lead to a miscalculation of the sign of higher contrasts, which may explain why the outliers in Fig. 1A all had low L−M contrast.

### L−M cone opponency and red/green color opponency

According to one popular theory, our color perception is defined by two opponencies: red *vs*. green, and blue *vs*. yellow [32], [33], [34]. It is now known that the *perceptual* color opponency is different from the cone opponency expressed in the retinogeniculate pathway [35]. Since the red-green axis is close to the L−M axis in color space [35], and the former is much more salient than the latter, one may wonder whether the two-way categorization intrinsic to color-naming systems might reflect the red-green color opponency or the L−M cone opponency.

To address this issue, we compared the direction of the red/green border in the isoluminant color plane with that of the “warm/cool” border. The isoluminant plane is defined by two cardinal axes [17], one representing the L−M cone contrast, the other representing the S cone contrast. Fig. 3 of De Valois et al. (1997) [35] showed that the red/green borders deviated from the S axis by various degrees, depending on the subject and the polarity of the S cone contrast. From that figure, we estimated that, for an average subject and with either polarity of S cone contrast, a straight line that would optimally separate the reddish and greenish colors deviates from the S axis by ∼9.6 degrees. This estimate was calculated after rescaling the two axes to match their contrasts. By contrast, the corresponding deviation of the “warm/cool” border is 0.75 degrees, as estimated by SVM analysis on the WCS data when they are represented in the color space formed by the above cardinal axes and a luminance axis. Accordingly, the “warm/cool” border is 12 times closer to the S axis than it is to the red/green border. Therefore, this border is more likely to coincide with the S axis, which corresponds to the reversal in the sign of the L−M contrast, than with the red/green border.

### The representation of color in V1

Most studies of color representation in primate V1 found that the majority of color-selective cells in V1 received mixed inputs from the two cone-opponent channels in the retinogeniculate pathway [36], [37], [38], [39], although the input from the L−M channel was dominant [40]. Consequently, compared to the lateral geniculate nucleus [17], V1 contains a more even distribution of cells that are tuned to various directions in color space. In studies that reported slightly uneven distributions, the most populated chromatic directions were between the L−M and S cardinal axes in the isoluminant plane [37], [38]. Consistent with these reports, our previous analysis of the data that were used in the present study has found a continuous systematic representation of color in an array of small regions in V1. Some of these regions corresponded to the response patches that were analyzed in the present study [18]. These regions were called hue maps because the response peaks within them were spatially organized according the stimulus hue. These hue maps coincided with the so-called color-preferring regions that responded preferentially to isoluminant chromatic stimuli, compared with achromatic stimuli. Because the color-preferring regions were found to be co-localized with the cytochrome oxidase blobs [41], [42], [43], the hue maps we found are likely to be co-localized with the blobs as well. The existence of hue maps in V1 was confirmed by a recent study using calcium-based two-photon microscopy [44]. That study found that in some regions around the cytochrome oxidase blobs, neurons were spatially organized according to their color tuning.

If there were no preference to the L−M axis among color selective cells in V1, how did this particular color direction seem to exert a stronger influence on color naming than did most of the other directions? Our study provides a possible answer to this question by demonstrating that the sign along the L−M axis is likely to determine the two-way clustering of the population responses in V1 hue maps. The cellular mechanism underlying this behavior of hue maps is unclear. One possibility is that hue maps are predominantly populated by the color-preferring cells reported by Johnson et al. [40], [45]. These cells are largely driven by opposite and balanced inputs from L and M cones, with only weak inputs from S cone [40]. Consequently, colors of opposite L−M contrasts are likely to activate two complimentary groups of color-preferring cells, which in turn produce two distinct clusters of response pattern as reported here. The weaker S-cone input is likely to have small influence on the response patterns without changing their two-way clustering. Therefore, our current finding is not at odds with our previous one regarding the spatially organized representation of the full hue gamut. The L−M sign and the hue of a stimulus can be decoded from the population activity of a hue map by different read-out mechanisms. In fact, by linking the response in hue maps with a fundamental constraint on color naming, our study corroborates the proposition we put forth in our previous report, namely, that hue maps play an important role in the color perception.

A related issue concerns the nonlinearity of the cortical response to color. Since many neurons in V1 combine cone inputs nonlinearly [38], [46], it seems surprising that both the two-way categorization of colors in WCS and the clustering of cortical responses in monkeys can be explained largely by a process that combines L- and M-cone contrasts linearly. One possibility is that the sign of the L−M cone contrast of a stimulus determines which one of the two neuronal populations in V1 will respond, and the magnitude of each neuron's response is determined nonlinearly by various cone contrasts. The two-way categorization or clustering under study here may simply reflect the first process.

Our *k*-means cluster analysis was carried out on the normalized optical signal instead of the raw one in the activation maps. The normalization consists of two steps. First, the average pixel value across a response patch in an activation map was subtracted from each pixel in the given patch. The resulting pixel value measures the *relative* signal across a response patch, instead of the *absolute* one that is measured in the original activation maps; Second, each pixel value was divided by the standard deviation of pixel values across the given patch. We note that the first step of the normalization is necessary for observing the reported relationship between the cortical responses and cone contrasts, whereas skipping the second step does not change our results qualitatively. The necessity of the first step is consistent with a previous report that the absolute optical signal in response to a stimulus comprises the signal elicited by the stimulus and the one associated with ongoing spontaneous activity [47]. Since the ongoing activity is coherent across an area that is larger than a typical hue map (>0.5 mm *versus* 0.2 mm across), its influence on the *relative* signal across a hue map is expected to be much smaller than that on the *absolute* signal. Therefore, it is likely that the stimulus-elicited signal is more correlated with the *relative* signal than it is with the *absolute* one. Both findings reported in the current and previous studies [18] were based on the analysis of the relative signal instead of the absolute one.

## Materials and Methods

### Ethics Statement

In the present study we reanalyzed data from experiments that have been reported previously [18]. All procedures were approved by the local Institutional Animal Welfare Committee, and were in full compliance with the NIH guidelines for the use of laboratory animals. Animals were housed according to **“Guide for the Care and Use of Laboratory Animals”** (Institute of Laboratory Animal Resources, Commission on Life Sciences, National Research Council, National Academy Press, Washington, D.C.). Details of the experimental procedures can be found in ref. [18]. Briefly, area V1 of four hemispheres from four anaesthetized macaque monkeys (*m. fascicuaris*) was studied with imaging of intrinsic optical signals. General anesthesia was induced by ketamine (10 mg/kg), and continued with a mixture of propofol (4 mg/kg/h) and sufentanil citrate (0.05 µg/kg/h). The depth of anesthesia was monitored by end-tidal CO_2_, blood pressure, and EEG. A bolus (0.1–0.5 cc) of the propofol/suentanil mixture was given whenever a sudden change in above measures was detected, or when a peak at alpha or higher bands appeared in the power spectrum of EEG.

### Calculation of cone contrasts of Munsell color chips

The spectral reflectance of the glossy Munsell color chips that were used in the WCS survey were downloaded from http://spectral.joensuu.fi. The spectral power distribution (SPD) of the reflected light from each chip was calculated by multiplying the chip's spectral reflectance with the spectrum of the CIE standard illuminant D65, assuming that the WCS tests were carried out under daylight.

L-, M-, and S-cone excitations were given by the dot product of the SPD and the cone fundamentals of Sharp and Stockman [48]. The L-cone fundamental was multiplied by a factor of 1.98 so that the sum of L- and M-cone fundamentals approximates the luminous efficiency function [49]. The cone contrasts of each chip were given by comparing the cone excitations of the given chip with those of the achromatic (gray) chip with a lightness value of 5.

### Visual stimulation for imaging experiments

**Figure 4:**
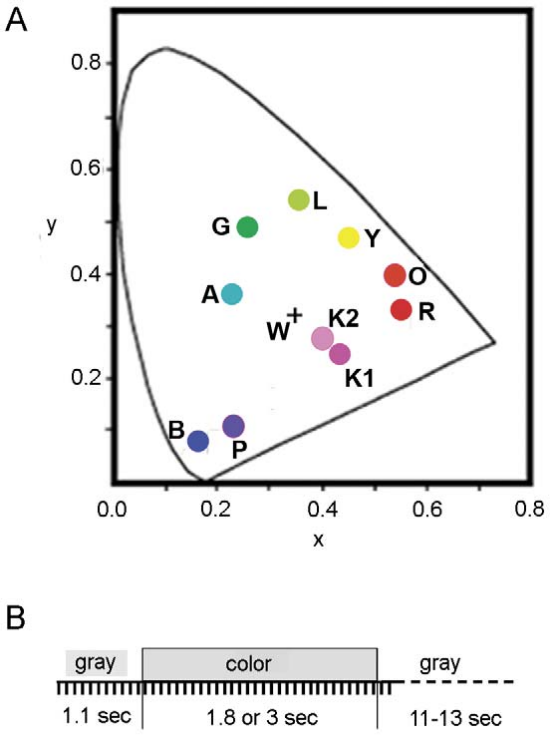
Visual stimuli and temporal order of experiments. **A**, CIE 1931-xy coordinates of the stimulus colors. R, red; O, Orange; Y, Yellow; L, Lime; G, green; A, aqua; B, blue; P, purple; K, pink; W, gray. **B**: Temporal sequence of stimulus presentation and data acquisition. Each tick mark indicates the time at which a cortical image was acquired. Adapted from ref [18] with permission.

Visual stimuli were spatially uniform color fields subtending 36×27° of visual angle. Nine to eleven isoluminant colors (9.5 cd/m^2^) were presented in each experiment, alternating with a uniform gray at the same luminance level as the tested colors. To compare the results from different experiments, the current study focused on nine stimulus colors that were used in all experiments. They were red, pink, purple, blue, aqua, green, lime, yellow, and orange. Their CIE1931-*xy* coordinates were (Fig. 4A): red (R) (0.55, 0.33), orange (O) (0.54, 0.40), yellow (Y) (0.45, 0.47), lime (L) (0.35, 0.54), green (G) (0.27, 0.49), aqua (A) (0.23, 0.36), blue (B) (0.16, 0.08), purple (P) (0.23, 0.12), pink (K1) (0.43, 0.24) or (K2) (0.38, 0.27), and gray (W) (0.32, 0.32) or (0.35, 0.32). Their chromaticities were close to those of basic chromatic colors (except for the color brown) in English, as identified by Berlin and Kay (1969) and Boyton et al. [50], [51]. With the additional colors lime and aqua, the stimulus set comprises all perceptually salient hues.

The SPDs of the display's phosphors were measured with a spectroradiometer (PR-650, Photo Research, Inc., CA). The SPD of each color (including the gray) was given by the dot product of the phosphor's SPDs and the phosphor intensity (gamma corrected) of the color. Cone excitations of a color were given by the dot product of its SPD and the cone fundamentals. The cone contrasts of each color were determined by comparing its cone excitations with those of the adapting gray.

### Procedure of optical imaging and data analysis

Details of the imaging procedure can be found in ref. [18]. Briefly, the intrinsic optical signal was recorded using a CCD camera with 652×492 pixels. The camera was focused 0–400 µm below the cortical surface, and took 10 frames/second at a resolution of 6.1 µm/pixel. The cortex was illuminated by 610 (+/−8) nm light from LEDs driven by a stabilized power supply.

During each imaging trial, 11 frames were taken before, 18 or 30 during, and 2 after the 1.8- or 3-second presentation of a stimulus, followed by a rest period of 11–13 seconds, during which the display presented a uniform gray (Fig. 4B). The animals were adapted to the same uniform gray before each experiment. An imaging block consisted of one imaging trial for each stimulus, including a control trial without color stimulation, presented in a pseudo-random order. Functional maps were derived from an experiment consisting of 50–51 imaging blocks.

For each imaging trial, we calculated the average of 11 pre-stimulation frames and the average of the last 7 frames (5 during-stimulation frames and 2 after-stimulation ones). They are called *pre-stimulation frames* and *response frames*, respectively. To reduce the artifacts caused by brain movement, all these frames in an experiment were spatially registered with the pre-stimulation frame of the first trial during the experiment. The registration was achieved by translating one frame relative the other until the Pearson correlation between them was maximized. Due to this operation, the frame size in the present study was reduced to 612×452 pixels.

To calculate a ‘single-condition’ activation map that represents the change in surface reflectance elicited by a stimulus, we compared the group of *pre-stimulation frames* with the group of *response frames* that were associated with the given stimulus. In the previous study [18], as well as most optical imaging studies, the conventional method of subtraction and normalization was used to derive the single-condition activation maps. In the present study, the activation maps were derived with a new method that is based on the statistical classification algorithm of the *Support Vector Machine* (SVM) [21], [25]. We have shown previously that this new method removes most of the artifacts associated with blood vessels and improves significantly the quality of the activation maps [25]. This improvement is evident when Fig. 2A and 2B are compared.

To identify cortical regions where response is greater than noise, the magnitude of noise in these activation maps was estimated from the map that was derived from the control trials in which the display was maintained at the adaptation gray. The same adaptation gray was also presented before and after color stimuli in all other trials. There was no response patch in the control map (Fig. 2E)– the variation in pixel value across the map is due to noise since the display was not changed during the control trials. Because response signal in intrinsic optical imaging is negative, we used the most negative value in the control map as the threshold to differentiate response signal from noise.

**Figure 5:**
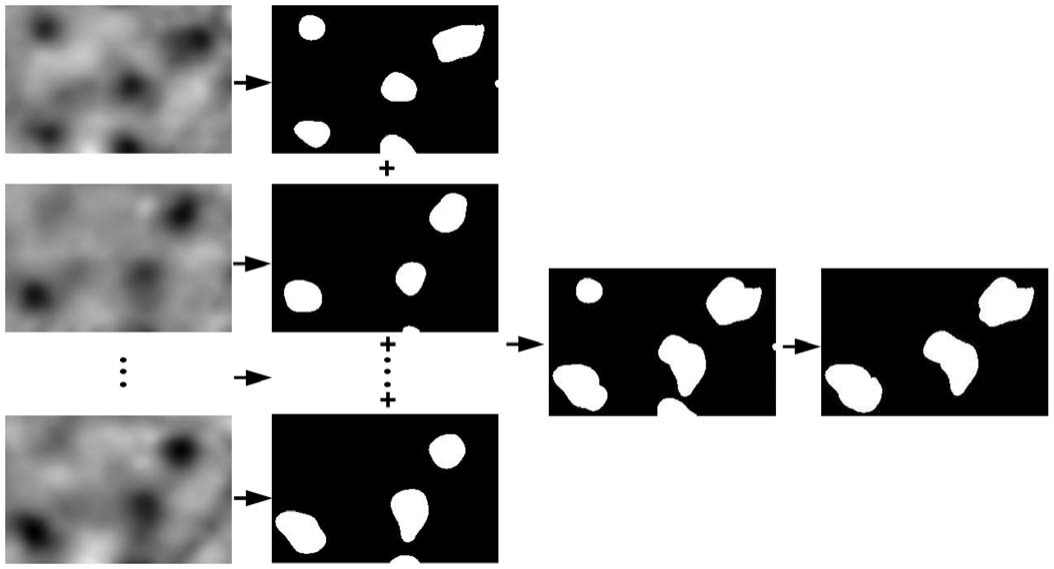
Identification of response patches in V1. The first column from the left shows a part of the activation maps associated with different stimulus colors. The three vertical dots stand for six more maps that are not shown due to space limitation. A threshold derived from the control map was used to identify the individual response regions in each activation map. These response regions are shown as the white patches in the second column from the left. The individual response regions elicited by various stimuli were combined to form the composite response patches, as shown in the third column from the left. The rightmost column shows the kind of composite response patches that were analyzed in the current study. Each of these patches meets both of the following criteria: 1) it encompasses individual response regions that are associated with five or more stimulus colors; 2) it doesn't extend to the map borders.

The threshold was then applied to each of the nine activation maps, to identify regions with response that was significantly above the noise. Nine maps of response regions will be generated, in which each pixel has a value 1 or 0 depending on whether or not the corresponding pixel in the activation maps has a value exceeding the threshold (for an example, see Fig. 5, the second column from the left). The individual response regions in these binary maps were then combined by the logic OR operation to form the composite response patches (Fig. 5, the third column from the left). To minimize the impact of noise, and maximize the number of the composite response patches for further analysis, we focused on composite response patches that are made of individual response regions of five or more stimulus colors (Fig. 5, the rightmost column). The composite patches consisting of less than five individual response regions were excluded because more than half of the stimuli failed to elicit above-noise response in the patch. Also excluded were the patches that extended beyond the map borders, due to incomplete information about their response distribution.

For each response patch of *N* pixels, the response to a color was represented by a vector *of N* elements, where each element is the value of a pixel within that patch in the corresponding activation map. Nine response vectors were obtained for each response patch. Each response vector was normalized to have zero mean and unit standard deviation. These nine vectors were subjected to the *k-*means cluster analysis (*k* = 2) [52], [53]. This analysis was implemented with the function k*means* in the Statistics Toolbox of Matlab (version 7.2, Mathworks, MA), which uses a two-phase iterative algorithm to minimize the sum of point-to-centroid Euclidean distances, summed over two clusters. Each point here represents a response vector in an *N*-dimension space. The centroid of a cluster is calculated as the element-wise mean across all cluster members.

### *k-*means cluster analysis

Details of the *k-*means algorithm can be found at http://www.mathworks.com/help/toolbox/stats/kmeans.html. Briefly, in the first phase, each iteration consists of reassigning points to their nearest cluster centroid, all at once, followed by recalculation of cluster centroids. Another iteration follows, based on the updated centroids. Two randomly chosen points are used as the initial centroids of the two clusters, the so-called *seeds*. This phase stops when additional iterations make no difference, and the outcome is used as the starting point for the second phase.

In the second phase, points are individually reassigned if doing so will reduce the sum of distances, and cluster centroids are recomputed after each reassignment. Each iteration during this phase consists of one pass through all the points. The second phase could converge to a local minimum. To locate the global minimum, we ran through the whole procedure with every possible pair of vectors as the seeds, and chose the run with the minimal sum of distances.

### Support Vector Machines (SVM) classification algorithm

Let c_i_ denote the group to which the vector X_i_ belongs. Each c_i_ has a value of either 1 or −1 for a two-way classification. A linear SVM classifier derives a separating hyperplane in the form of
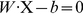
(2)


The SVM algorithm defines two hyperplanes that are parallel to the separating one (Eq. 2), one on each side of the separating hyperplane with a equal distance (*d*) to the latter. Each of these two parallel hyperplane passes at least one member vector of one group, and ideally there is no vector between them. The SVM algorithm searches for a specific hyperplane with associated parallel hyperplanes that are maximally separated.

The weight vector *W* in Eq. 2 is orthogonal to the hyperplane, and has a length of 1/*d*. Therefore *W* is derived by the following optimization:
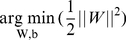
(3)subject to the condition that 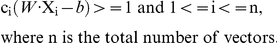



This algorithm applies to cases where the two groups can be completely separated by a hyperplane. Such cases are called linearly separable.

If the two groups are not linearly separable, the weight vector W is derived by the following optimization:
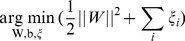
(4)subject to the condition that
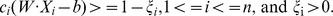



The SVM classification was implemented with a *Matlab* program provided by Chang and Lin (LIBSVM: a library for support vector machines, 2001; available at http://www.csie.ntu.edu.tw/~cjlin/libsvm/). In order to use the weight vector *W* to rank the input variables by their importance in the classification [22], and to use information criteria to select input variables that contributed to a classification [23], each input variable in the vector X was normalized to have a mean value of 0 and a unit standard deviation before an SVM classification was performed. The weights in the resultant classifier represented the weights of the normalized variables. These weights were then converted to represent the weights of the original variables. The intercept, *b*, of the hyperplane was also converted to represent the value in the space of the original variables. The converted values are reported in the hyperplane equations of the Results section.

#### Cross-validation

The performance of the SVM classification was evaluated by cross-validation. The entire dataset was divided into n equal parts for n-fold cross-validation. For each part, the data in the remaining n−1 parts were used to train the SVM classifier, and those in the given part were used to test the performance of the classifier. The percentage of the correct classifications across the test data, or the accuracy, was calculated. These accuracies, one for each part of the data, were then averaged to give the accuracy that was associated with the n-fold cross-validation of the classification.

Two information criteria for an SVM classifier were calculated according to Claeskens *et al.* (2008) [23]:
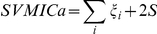


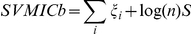
where *ξ_i_* is determined by Eq. [4], *S* denotes the number of input variables of the classifier, and *n* denotes the size of the training set.
